# The use of a modified posterior approach (SPAIRE) may be associated with an increase in return to pre-injury level of mobility compared to a standard lateral approach in hemiarthroplasty for displaced intracapsular hip fractures: a single-centre study of the first 285 cases over a period of 3.5 years

**DOI:** 10.1007/s00068-022-02047-1

**Published:** 2022-07-25

**Authors:** John Charity, Susan Ball, Andrew J. Timperley

**Affiliations:** 1 The Hip Unit, Princess Elizabeth Orthopaedic Centre, Royal Devon University Healthcare NHS Foundation Trust, Barrack Road, Exeter, EX2 5DW UK; 2 NIHR Applied Research Collaboration (ARC) South West Peninsula, University of Exeter Medical School, St Luke’s Campus, Exeter, EX1 2LU UK

**Keywords:** Hip hemiarthroplasty, Posterior approach, Tendon sparing, SPAIRE, Mobility

## Abstract

**Background and purpose:**

A tendon-sparing modification of the posterior approach to the hip joint was introduced in the specialist hip unit at our institution in 2016. The SPAIRE technique—acronym for “Saving Piriformis And Internus, Repair of Externus” preserves the insertions of gemellus inferior, obturator internus, gemellus superior and piriformis intact. We compare the results of the first 285 hip hemiarthroplasty patients, unselected but preferentially treated by our hip unit surgeons using the SPAIRE technique, with 567 patients treated by all orthopaedic surgeons (including the hip unit) in the department over the same 3.5 year period using the standard lateral approach. We report length of stay, return to pre-injury level of mobility, place of residence and mortality at 120 days.

**Patients and methods:**

The review included all hemiarthroplasty patients. Pre-fracture mobility and place of residence, surgical approach, grade of senior surgeon in theatre, stem modularity, acute and overall length of stay, mobility, place of residence, re-operations and mortality at 120 days were recorded. Data were obtained from the National Hip Fracture Database that included a telephone follow-up at 120 days and from electronic patient records.

**Results:**

The odds of returning to pre-injury level of mobility were higher in the SPAIRE technique group than in the standard lateral group; adjusted odds ratio (95% confidence interval (CI)) 1.7 (1.1 to 2.7, *p* = 0.01).

**Interpretation:**

When used in hip hemiarthroplasty, the SPAIRE technique appears safe and may confer benefit in maintaining the pre-injury level of mobility over the standard lateral approach.

## Introduction

Displaced intracapsular hip fracture patients are treated with arthroplasty, most frequently being a hemiarthroplasty [1]. Mobility and functional independence have been reported to be the main priority for hip fracture patients [2].

The National Institute for Health and Care Excellence (NICE) from the United Kingdom recommends that surgeons consider the lateral in favour of the posterior approach when inserting a hemiarthroplasty, but acknowledges that evidence for this recommendation is of very poor quality, being based on only two studies [3–5].

Evidence from an observational study from the Norwegian Hip Fracture Register of over 20,000 patients suggests better patient-related outcome measures of pain, patient satisfaction and health-related quality of life with the standard posterior approach when compared with the lateral approach although the recorded dislocation rate was much higher in the posterior approach group [6]. Dislocation, although relatively rare can lead to catastrophic consequences in this frail cohort of patients [7–9].

In this paper we describe a reproducible tendon-sparing modification of the posterior approach using the muscle interval between gemellus inferior and quadratus femoris leaving the insertions of gemellus inferior, obturator internus, gemellus superior and piriformis intact—the SPAIRE technique (acronym for “Saving Piriformis And Internus, Repair of Externus”). This has been adopted by the surgeons at our specialist hip unit when performing hip hemiarthroplasty for displaced intracapsular fractures. In 2020, the SPAIRE technique was also adopted in 5.7% of all hemiarthroplasties performed in Norway, as shown in the 2021 annual report of the Norwegian hip fracture database [10].

The aim of this article is to report on details of the SPAIRE technique with results on the first 285 hemiarthroplasty cases performed at our unit, comparing return to pre-injury level of mobility of these patients at 120 days post-admission with that of hemiarthroplasty patients operated on over the same period for whom the standard lateral approach was used.

## Patients and methods

Data were obtained from the NHFD that included a telephone follow-up at 120 days, and we searched our hospital electronic patient record system for all patients undergoing hip hemiarthroplasty between 28 September 2016 and 31 March 2020. We recorded return to pre-injury level of mobility at 120 days post-admission (routinely recorded on the NHFD database as one of the following categorical variables: “freely mobile without aids”, “mobile outdoors with one aid”, “mobile outdoors with two aids or frame”, “some indoor mobility but never goes outside without help”, and “no functional mobility”), length of stay, pre- and post-operative place of residence, complications requiring re-operations including closed manipulation of dislocated joints and mortality within 120 days. One hundred and twenty-day follow-up rates recorded on the NHFD for our hospital (for all hip fracture types) were over 97% over the 4-year study period (97.1% in 2019, 98% in 2018, 97.9% in 2017 and 99.3% in 2016).

### Operative technique (standard lateral approach)

The patient is positioned in the lateral position with both hips in slight flexion and neutral rotation. The draping process includes making a leg bag to prevent contamination of the operated limb during femoral preparation. A skin incision is made over the lateral aspect of the greater trochanter. Dissection is made down to the fascia lata, which is incised distally to allow blunt splitting of the fibres of gluteus maximus proximally, exposing the trochanteric bursa as well as the tendinous cuff that originates from the fibres of gluteus medius inserting onto the greater trochanter. The cuff is sharply incised along its anterior and proximal borders, leaving the posterior third of the trochanteric attachment intact ensuring a tendinous edge is left on each side of the incised cuff allowing for adequate closure at the end of the procedure. Fibres of gluteus minimus are divided along with the anterior capsule exposing the anterior aspect of the femoral neck and fracture. Femoral neck osteotomy is carried out obliquely allowing the femoral head to be retrieved. The acetabular cavity is checked for intact cartilage and protected with a saline-soaked swab. The limb is positioned inside the leg bag to allow access to the femoral canal. After adequate preparation of the proximal femur, a prosthesis of correct size, offset and position is implanted. The saline-soaked swab is removed from the acetabular cavity prior to the final reduction. The anterior capsule and fibres of gluteus minimus are closed with non-absorbable suture followed by reattachment of the cuff of gluteus medius tendon onto the greater trochanter.

### Operative technique (SPAIRE)

**Fig. 1:**
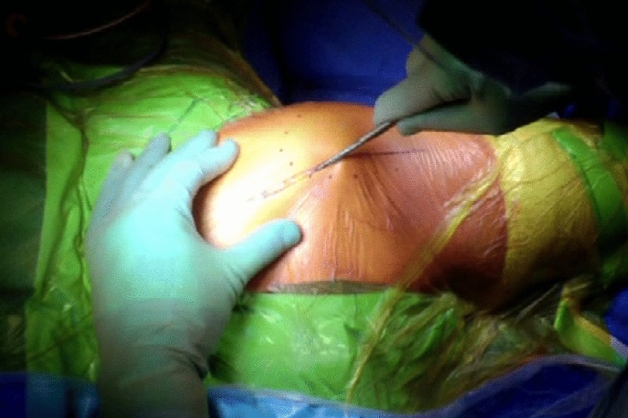
A skin incision is made between the level of gluteus maximus insertion on the femur, along the posterior border of the greater trochanter, curving gently posteriorly in the region proximal to the tip of the greater trochanter

**Fig. 2:**
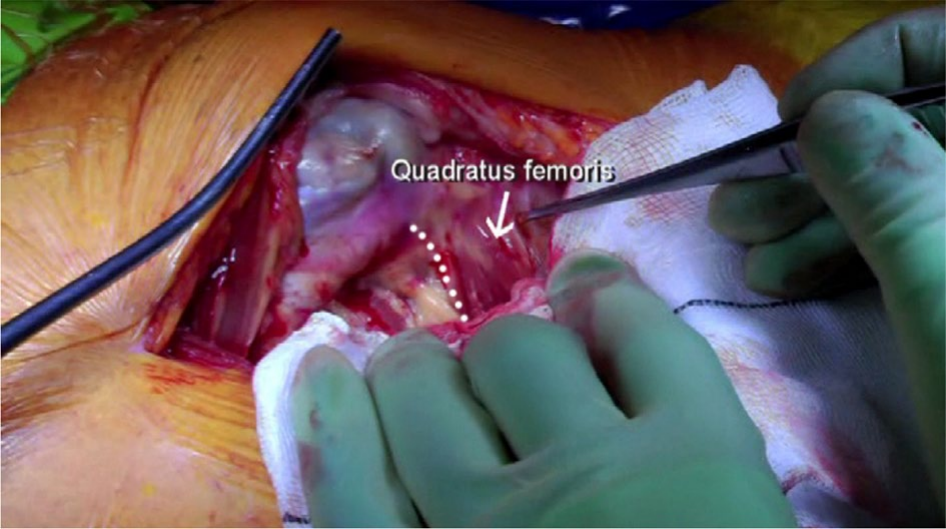
The interval between the insertions of quadratus femoris and gemellus inferior muscles onto the posterior aspect of the greater trochanter is identified by blunt dissection

**Fig. 3:**
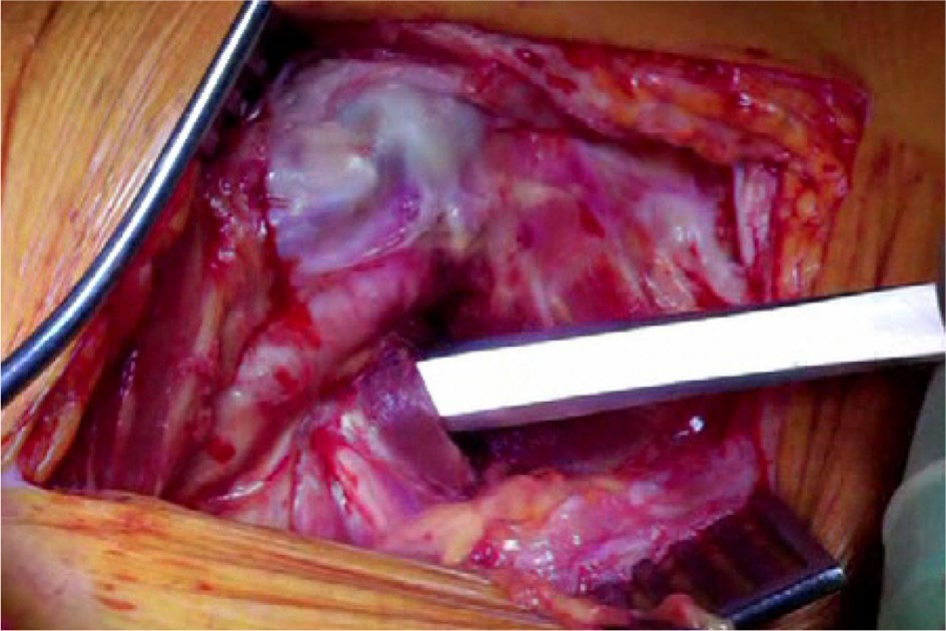
A plane is developed by passing a periosteal elevator between the capsule and the proximal short external rotators (namely gemellus inferior, obturator internus, capsule gemellus superior and piriformis)

**Fig. 4:**
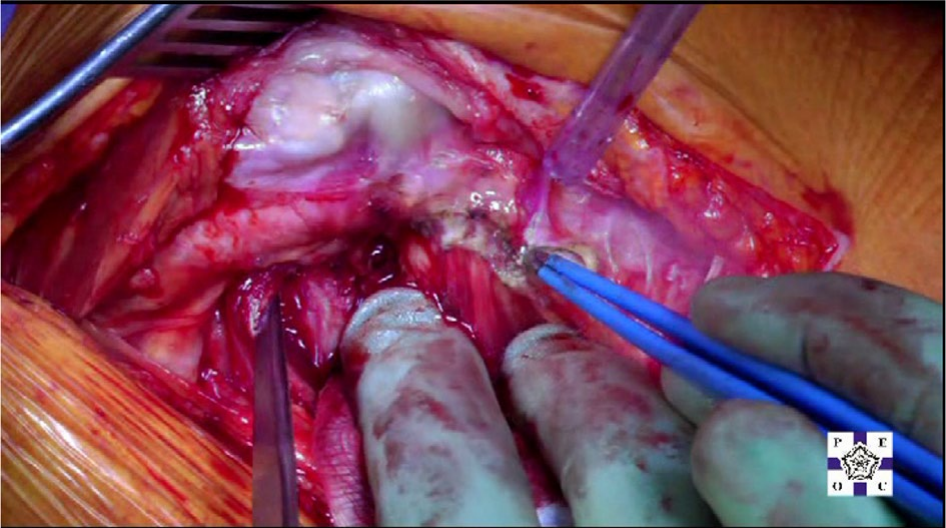
The proximal half of the quadratus femoris muscle insertion is then divided along with the insertion of obturator externus and the posterior capsule insertion onto the posterior aspect of the femoral neck

**Fig. 5:**
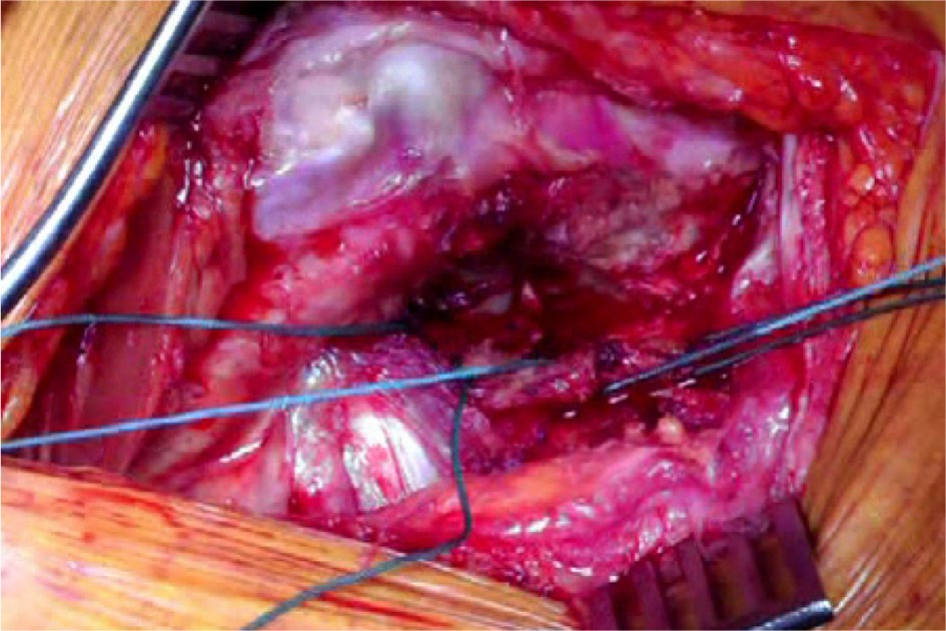
The capsule tagged in three distinct points using a strong non-absorbable suture: proximally, distally along with the adjacent tendon insertion of obturator externus and a third point midway between both

The patient is positioned in the lateral position with both hips in slight flexion and neutral rotation. A skin incision is made from the area where the tendon of gluteus maximus inserts on the femur, along the posterior border of the greater trochanter to reach the area proximal to its tip (Fig. 1). Dissection is made down to the fascia lata, which is incised distally to allow blunt splitting of the fibres of gluteus maximus proximally, exposing the trochanteric bursa and posterolateral aspect of the greater trochanter. The interval between the insertions of quadratus femoris and gemellus inferior muscles onto the posterior aspect of the greater trochanter is identified by blunt dissection (Fig. 2), revealing the trochanteric branches of the medial circumflex artery which are cauterised and divided exposing the posterior capsule. The sciatic nerve is identified posterior to the short external rotators. Tension is then taken off the short external rotators by an assistant lifting the knee to a position of neutral hip abduction. A plane is developed by passing a periosteal elevator between the capsule and the proximal short external rotators (namely gemellus inferior, obturator internus, gemellus superior and piriformis—Fig. 3). This muscle group is elevated with a slim retractor. The proximal half of the quadratus femoris muscle insertion is divided along with the insertion of obturator externus and the posterior capsule insertion onto the posterior aspect of the femoral neck (Fig. 4). The proximal extension of the posterior capsulotomy is completed taking care not to damage the proximal short external rotators which are retracted, or the labrum. The capsule is tagged in three distinct points using braided non-absorbable suture (Ethibond number 2—Ethicon, Sommerville, New Jersey, USA) proximally, distally along with the adjacent tendon insertion of obturator externus and a third point midway between both (Fig. 5). The tagged capsule is lifted and retracted, acting as a protective layer over the sciatic nerve.

**Fig. 6:**
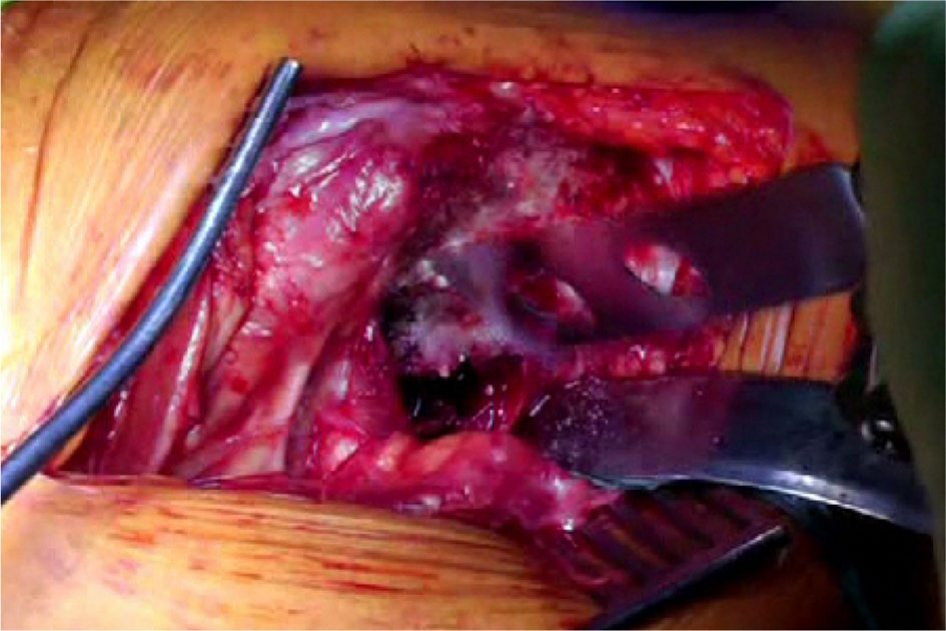
Inferior retractor placed on the medial surface of the femoral neck allowing for a safe femoral neck osteotomy

The hip is flexed further and internally rotated to 90°. This manoeuvre completes the fracture displacement and exposes the femoral neck beneath the intact group of short external rotators. An inferior retractor is placed on the medial surface of the femoral neck allowing for a safe osteotomy of the femoral neck (Fig. 6). Loose bone fragments and redundant capsular attachments are removed. The femoral head is extracted using a tapered cork-screw, revealing the acetabular cavity for inspection of its articular surface and labrum, and allowing removal of any remaining bony fragments. A small swab soaked in saline in placed in the acetabulum for protection of its articular surface.

**Fig. 7:**
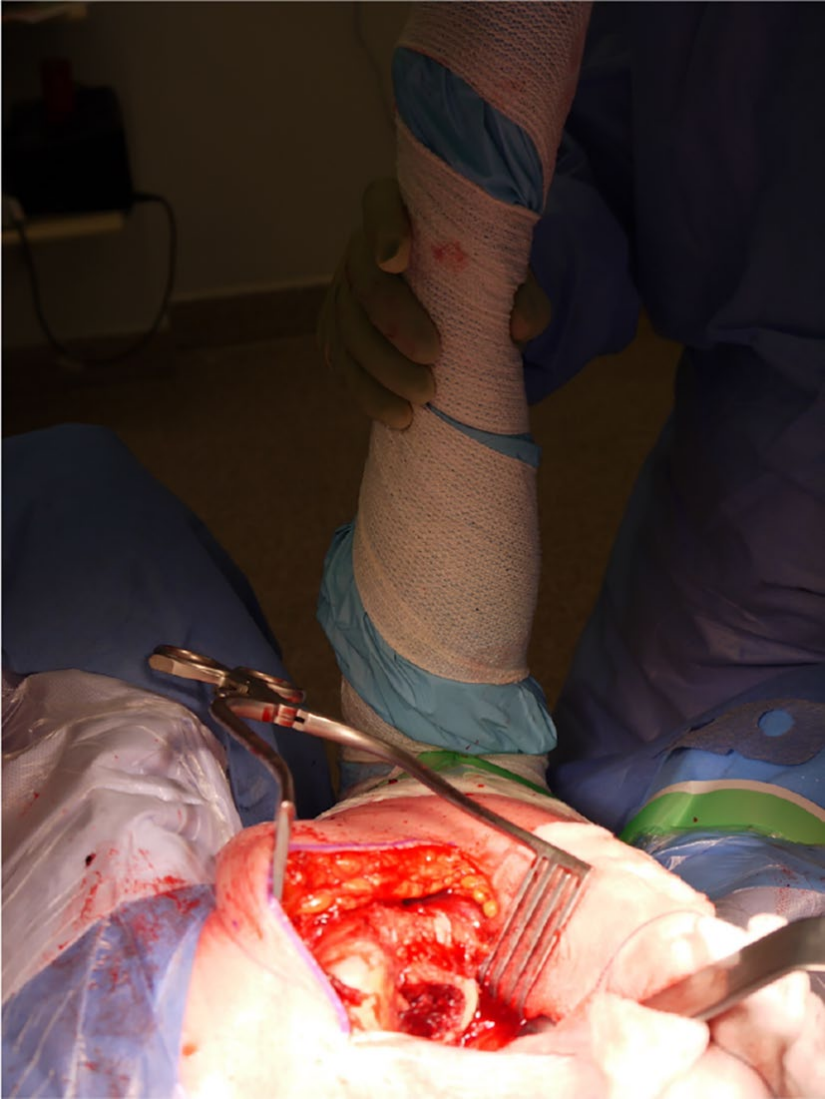
Osteotomised femoral neck ready for access and preparation of the proximal femoral canal. Note the insertions of the proximal short external rotators on the medial surface of the greater trochanter, positioned anterior to the entry point for instrumentation of the femoral canal

With a saline-soaked swab protecting the posterior soft tissues and skin edge of the surgical wound, a femoral elevator is placed under the anteromedial aspect of the osteotomised femoral neck. The hip is flexed to near 90° and held in 90° of internal rotation by the assistant. This position exposes the cut surface of the femoral neck ready for access and preparation of the proximal femoral canal (Fig. 7). The insertions of the proximal short external rotators that are left intact in this technique are located anteriorly on the medial surface of the greater trochanter as demonstrated by Ito et al. and do not obstruct access to the femoral canal, so instrumentation can be carried out as normal [11].

**Fig. 8:**
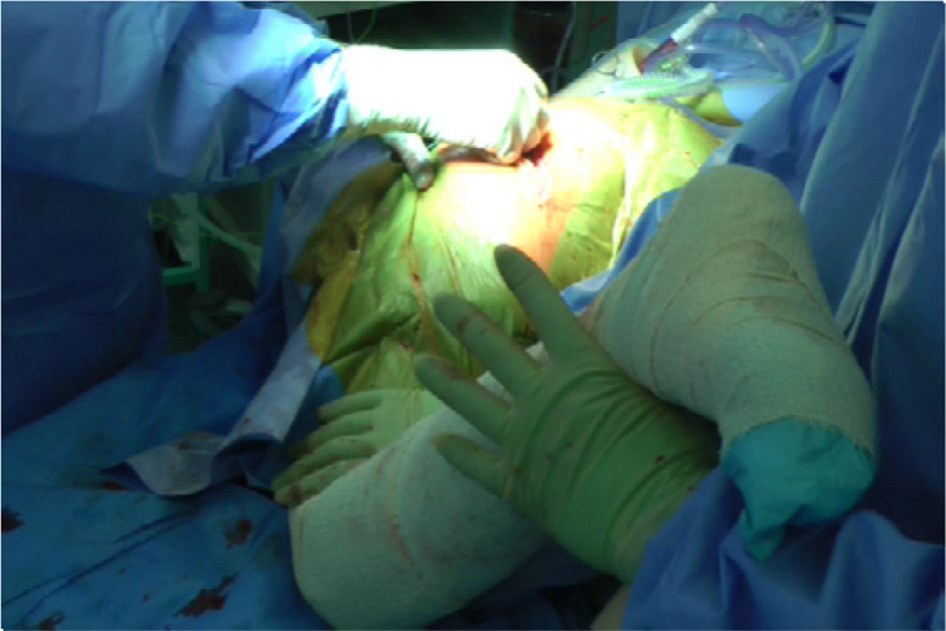
Test of stability in flexion–adduction–internal rotation (FAIR) revealing the intact proximal short internal rotators, which tighten up preventing posterior dislocation

A definitive femoral prosthesis of correct size, femoral offset and position is implanted as per the corresponding operative technique and pre-operative planning. The swab soaked in saline is removed from the acetabulum and the hip reduced. A test of stability in flexion–adduction–internal rotation (FAIR) reveals the intact proximal short internal rotators, which tighten up in FAIR preventing posterior dislocation (Fig. 8). The hip is also checked for stability throughout its arc of motion including extension–abduction–external rotation (EAER), which is stabilised by the intact anterior capsule.

**Fig. 9:**
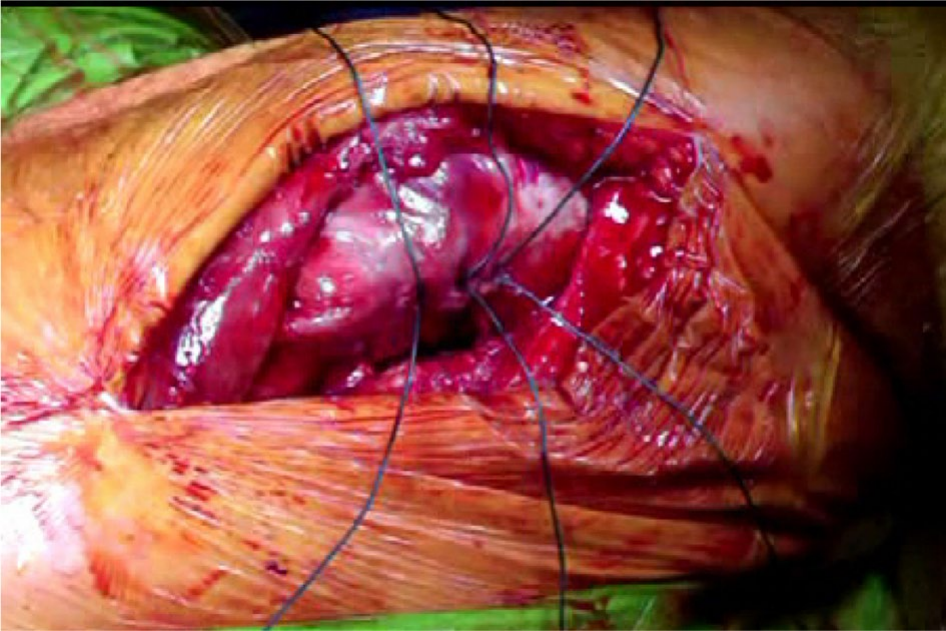
Prior to closure, two 2.5 mm drill holes are made on the posterior aspect of the greater trochanter at the level of each stay suture, which are passed through. A third suture passed through the gluteal tendon insertion is also shown

Prior to closure, two 2.5 mm drill holes are made on the posterior aspect of the greater trochanter at the level of each stay suture, which are passed through. A stay suture may be passed through the gluteal insertion on the greater trochanter. Figure 9 shows three stay sutures used, one of which passed through the gluteal tendon insertion. The stay sutures are tied accordingly with each knot positioned over its respective hole avoiding prominence. Each suture is then trimmed short once tied. Thorough irrigation with saline is followed by closure of fascia, fat and skin. Post-operative instructions allow for full weight bearing as able as soon as the anaesthetic wears off and there are no functional restrictions whatsoever.

A video demonstrating this technique is available on VuMedi, a medical education website (www.vumedi.com) [12].

### Analyses

Patient demographic characteristics are summarised for each surgical approach group, in terms of numbers and percentages for categorical variables and means and standard deviations or medians and interquartile ranges for continuous variables. The odds of returning to pre-injury level of mobility are compared between both groups using logistic regression and results reported as adjusted odds ratio (OR) with 95% confidence interval (CI) and *p* value. Similarly, further analyses use logistic regression to compare the odds of returning to pre-injury place of residence from ward and Trust discharge, and of being resident at pre-injury place of residence at 120 days follow-up. Ward and total length of stay are compared between both groups using linear regression and results reported as adjusted difference in means with 95% CI and *p* value. Mortality within 120 days is compared using Cox regression, and an adjusted hazard ratio (HR), 95% CI and *p* value reported. All analyses are adjusted for patient sex, age, pre-injury level of mobility, pre-injury place of residence, AMTS, ASA, type of operation and grade of senior surgeon present. Unadjusted analyses are also reported. All analyses were conducted in R [13].

## Results

285 cases using the SPAIRE technique were performed from 28 September 2016 to 31 March 2020. In the same period, 567 cases using the lateral approach were performed by all surgeons involved with trauma care at our department. 3 cases performed by a single surgeon using a modified posterior approach in which obturator internus was deliberately divided but piriformis preserved, were excluded from the analyses presented in this paper. Only 1 patient could not be contacted at 120 days so was lost to follow-up.

Table 1 summarises the population demographics.

**Table 1:** Demographic characteristics of patients, by surgical approach

Characteristic	SPAIRE (*N* = 285)	Lateral (*N* = 567)
Sex, *n* (%)		
Female	181 (63.5)	385 (67.9)
Male	104 (36.5)	182 (32.1)
Age in years, mean (SD)	85.61 (7.4)	85.44 (7.7)
Pre-fracture mobility, *n* (%)		
Freely mobile without aids	74 (26.0)	125 (22.0)
Mobile outdoors with one aid	78 (27.4)	135 (23.8)
Mobile outdoors with two aids or frame	31 (10.9)	76 (13.4)
Some indoor mobility but never goes outside without help	102 (35.8)	230 (40.6)
No functional mobility	0	1 (0.2)
Pre-fracture place of residence, *n* (%)		
Own home/sheltered housing	231 (81.1)	436 (76.9)
Residential care	34 (11.9)	90 (15.9)
Nursing care	20 (7.0)	41 (7.2)
Pre-operative AMTS, median (IQR)	9 (5 to 10)	8 (4 to 10)
Pre-operative AMTS, *n* (%)		
0–7	110 (38.6)	246 (43.4)
8–10	172 (60.4)	315 (55.6)
Not reported	3 (1.1)	6 (1.1)
ASA grade, *n* (%)		
1	2 (0.7)	5 (0.9)
2	56 (19.6)	104 (18.3)
3	201 (70.5)	411 (72.5)
4	26 (9.1)	45 (7.9)
5	0	2 (0.4)

SPAIRE: Saving Piriformis And Internus, Repair of Externus; AMTS: Abbreviated Mental Test Score; ASA: American Society of Anaesthesiologists

Table 2 describes the two surgical approach groups in terms of the level of surgeon seniority present during surgery and the type of implant used. A cemented Exeter stem was used in every case in this cohort, being either a monoblock Exeter Trauma Stem (ETS—Stryker Corporation, Kalamazoo, Michigan, USA) or a bipolar modular construct using the Exeter V40 stem with an Orthinox stainless steel head in a Universal Head Bipolar System (UHR) bipolar head component (Stryker Corporation, Kalamazoo, Michigan, USA).

**Table 2:** Operation characteristics, by surgical approach

	SPAIRE (*N* = 285)	Lateral (*N* = 567)
Grade of senior surgeon present, *n* (%)		
Consultant level	253 (88.8)	470 (82.9)
ST3+	32 (11.2)	97 (17.1)
Stem type, *n* (%)		
Exeter V40 stem with UHR bipolar head	128 (44.9)	128 (22.6)
Exeter ETS monoblock stem	157 (55.1)	439 (77.4)

SPAIRE: Saving Piriformis And Internus, Repair of Externus; ST3+: Specialty Trainee year 3 or above; ETS: Exeter Trauma Stem (Stryker Corporation, Kalamazoo, Michigan, USA); UHR: Universal Head Bipolar System (Stryker Corporation, Kalamazoo, Michigan, USA)

Table 3 shows results of analyses comparing the outcomes measured between SPAIRE and lateral approaches. Data on 120-day level of mobility were available for 252 of the 285 (88.4%) SPAIRE and 460 of the 567 (81.1%) lateral procedures. The odds of returning to pre-injury level of mobility were higher in the SPAIRE group than in the lateral group, adjusted OR (95% CI) 1.7 (1.1 to 2.7), *p* = 0.01. Survival within 120 days was improved in the SPAIRE group, compared to the lateral group, adjusted HR for mortality (95% CI) 0.6 (0.4 to 0.9), *p* = 0.02. There was little evidence of an effect of surgical approach on other outcomes measured.

**Table 3:** Comparisons of outcomes between SPAIRE and lateral approach groups

Outcome	SPAIRE	Lateral	Statistic	Estimate (unadjusted)*	Estimate (95% CI) adjusted**	*p* value**
120-day mobility the same as pre-fracture mobility—based on 252 SPAIRE and 460 lateral^†^	*n* (%) 139 (55.2)	*n* (%) 227 (49.3)	Odds ratio	1.3	1.7 (1.1 to 2.7)	0.01
Mortality (within 120 days)	*n* (%) 33 (11.6)	*n* (%) 109 (19.2)	Hazard ratio	0.6	0.6 (0.4 to 0.9)	0.02
Ward length of stay, in days	Median (IQR), mean (SD) 9 (7 to 13), 10.6 (6.2)	Median (IQR), mean (SD) 10 (7 to 13), 11.7 (8.2)	Difference in means	− 1.1	− 1.0 (− 2.1 to 0.1)	0.08
Overall length of stay, in days	Median (IQR), mean (SD) 12 (8 to 25), 18.2 (15.06)	Median (IQR), mean (SD) 14 (8 to 26), 19.9 (16.9)	Difference in means	− 1.8	− 2.0 (− 4.3 to 0.2)	0.07
Ward discharge destination the same as pre-fracture residence—based on 278 SPAIRE and 537 lateral^††^	*n* (%) 154 (55.4)	*n* (%) 276 (51.4)	Odds ratio	1.2	1.3 (0.9 to 1.8)	0.1
Trust discharge destination the same as pre-fracture residence—based on 276 SPAIRE and 530 lateral^†††^	*n* (%) 231 (83.7)	*n* (%) 430 (81.1)	Odds ratio	1.2	1.1 (0.8 to 1.7)	0.5
120 day residence the same as pre-fracture residence—based on 252 SPAIRE and 460 lateral^†^	*n* (%) 202 (80.2)	*n* (%) 384 (83.5)	Odds ratio	0.8	0.8 (0.5 to 1.3)	0.4

SPAIRE: Saving Piriformis And Internus, Repair of Externus

^*^Model including surgical approach only

^**^Model including surgical approach, adjusting for sex, age, pre-fracture mobility, pre-op place of residence, AMTS (cognition—binary), ASA (comorbidities—3 categories), type of operation and grade of senior surgeon present

^†^Data on mobility and place of residence at 120 days post-surgery were not available for 33 (died before 120 days) SPAIRE and 107 (106 died before 120 days; 1 uncontactable) lateral participants

^††^7 SPAIRE and 30 lateral participants died before ward discharge and are excluded from this analysis

^†††^9 SPAIRE and 37 lateral participants died before trust discharge and are excluded from this analysis

Complications requiring re-operations in the entire series included 3 peri-prosthetic fractures (0.4%—2 cases from the lateral group treated with open reduction and internal fixation and 1 case from the SPAIRE group treated with revision of the femoral component). There were 3 peri-prosthetic infections (0.4%—1 case from the lateral group treated with washout and debridement only, 1 case from the lateral group treated with debridement and implant retention and 1 case from the SPAIRE group treated with Girdlestone excision arthroplasty). 4 dislocations (4 patients) occurred in the whole cohort (0.5%—3 cases from the lateral group (0.5%) treated with conversion to total hip replacement and 1 case from the SPAIRE group (0.4%) treated with closed manipulation under anaesthetic and no recurrence). There was 1 traumatic wound dehiscence in a case from the lateral group treated with washout and closure. There were no cases of nerve palsy.

## Discussion

In 2012, Han et al. reported a reduced incidence of dislocation using a minimally invasive posterior approach in patients suffering a neurological disorder. The authors described a modification by which piriformis, gemellus superior and obturator internus were left intact [7]. In their description, a conventional capsule repair was carried out prior to closure. Modifications in surgical technique have been previously attempted to improve joint stability after hemiarthroplasty in patients at increased risk as a result of a specific comorbidity [14]. The SPAIRE technique for total hip arthroplasty was reported by Hanly et al. [15].

The SPAIRE technique in hip hemiarthroplasty was first performed on 28 September 2016 by the senior author. An initial report on the first 25 hemiarthroplasty cases using the SPAIRE technique at our institution suggested the technique was feasible and safe to be used [16].

In a cadaveric study, Vaarbakken et al. demonstrated the importance of the short external rotators (namely the piriformis, gemellus superior, obturator internus and gemellus inferior muscles) in hip function, acting as the main extensor and abductor muscle group of the flexed hip (i.e. when the hip is in a flexed position of up to 90°). This is of particularly significant importance for movements such as rising from a chair and climbing stairs. The same authors named these four muscles *quadriceps coxa* due to their synergistic action on movements of the hip and for attaching as a same group onto the greater trochanter [17]. By preserving these important muscles along with gluteus medius and minimus, the SPAIRE technique may facilitate better recovery in hemiarthroplasty patients. This contrasts with the standard lateral approach, where a significant proportion of the largest muscle group of the hip, gluteus medius and minimus are split or divided, potentially impacting on hip function. We believe the combination of this muscle sparing approach with an enhanced capsular repair at the end of the procedure provide sufficient stability to enable patients to fully weight bear and mobilise with no restrictions whatsoever after surgery, the same as for the standard lateral approach. By preserving the short external rotators along with a posterior repair that includes capsule and obturator externus, the SPAIRE technique may reduce the dislocation rate associated with the standard posterior approach whilst potentially improving functional outcomes for this frail group of patients. The experience of our hip unit with this tendon-preserving technique has been positive, and although objective measurements have not been possible in this retrospective study, we have not observed an increase in surgical time or blood loss when compared to the standard lateral approach.

This report has limitations. It is an observational study of only 852 cases. Although the patient population in this study may not be large enough to capture significant differences between each group for rare events such as dislocation, infection or neurovascular injury, the single episode of a traumatic dislocation in the SPAIRE group (0.4%) versus three non-traumatic dislocation episodes in the lateral approach group (0.5%) and no reported neurovascular injury are reassuring findings and do not raise concerns of possible harm to patients associated with this new technique. While the majority of operations across both series were carried out in the presence of consultant level surgeons, the SPAIRE group comprised of hip subspecialty consultant level presence in 88.8% of cases, in contrast to involvement of consultant level from all orthopaedic subspecialties (including hip) in the lateral group in 82.9% of cases. Being a modification of the posterior approach, the SPAIRE technique is more likely to be adopted initially by hip subspecialty surgeons who are more familiar with that approach. However, specialist hip consultant level presence at surgery in the SPAIRE group is unlikely to explain the improved rates of return to pre-injury level of mobility and mortality observed in that group. No patients were selected for referral to the hip team; patients had their operations under the care of the orthopaedic consultant on-call, usually on the first available list after their admission as recommended by NICE guidance on hip fracture care [3]. A higher proportion of constructs using modular stems with a bipolar head were used in the SPAIRE group (44.9% vs. 22.6% in the lateral group). This construct is used in preference to a monoblock stem by the hip unit specialist surgeons to match individual patient anatomy in situations when it is felt that a monoblock stem will not meet this requirement. However, neither stem modularity nor the use of a bipolar head construct are likely to explain the improved rates of return to pre-injury level of mobility and mortality observed in the SPAIRE group. There is no evidence in the literature that either of these improve mobility or mortality rates in hip hemiarthroplasty patients within 120 days [18–20]. The SPAIRE approach has significantly better clinical outcomes than the lateral approach in this study. However, this is not to say that the SPAIRE approach is superior to the conventional posterior approach with regards to function. A comparison of the SPAIRE approach with the conventional posterior approach was not possible in this retrospective cohort as not used at our department.

Following the recommended stages of surgical innovation of the IDEAL framework a multicentre randomised controlled trial sponsored by our institution and funded by the National Institute for Health Research (NIHR) is under way [21]. Currently in its recruitment phase, the HemiSPAIRE study aims to compare the SPAIRE and the standard lateral approaches in hip hemiarthroplasty with regards to function.

## Conclusion

When treating displaced intracapsular fracture patients with hip hemiarthroplasty, the SPAIRE approach appears safe and may provide not only joint stability but also superior functional recovery, as demonstrated by an increase in return to pre-injury level of mobility compared to that of a standard lateral approach. However, further research is required to conclude any potential benefits. The SPAIRE technique is routinely used for hemiarthroplasty by all hip specialist surgeons and trainees at our unit.
